# Tumor mutational burden assessment in non-small-cell lung cancer samples: results from the TMB^2^ harmonization project comparing three NGS panels

**DOI:** 10.1136/jitc-2020-001904

**Published:** 2021-05-07

**Authors:** Javier Ramos-Paradas, Susana Hernández-Prieto, David Lora, Elena Sanchez, Aranzazu Rosado, Tamara Caniego-Casas, Nuria Carrizo, Ana Belén Enguita, María Teresa Muñoz-Jimenez, Borja Rodriguez, Urbicio Perez-Gonzalez, David Gómez-Sánchez, Irene Ferrer, Santiago Ponce Aix, Ángel Nuñez Buiza, Pilar Garrido, José Palacios, Fernando Lopez-Rios, Eva M Garrido-Martin, Luis Paz-Ares

**Affiliations:** 1 H12O-CNIO Lung Cancer Clinical Research Unit, Health Research Institute Hospital 12 de Octubre (imas12) / Spanish National Cancer Research Center (CNIO), Madrid, Spain; 2 Spanish Center for Biomedical Research Network in Oncology (CIBERONC), Madrid, Spain; 3 Pathology-Targeted Therapies Laboratory, HM Sanchinarro University Hospital, Madrid, Spain; 4 Scientific Support Unit, Health Research Institute Hospital 12 de Octubre (imas12), Madrid, Spain; 5 Spanish Center for Biomedical Research Network in Epidemiology and Public Health (CIBERESP), Madrid, Spain; 6 Faculty of Statistical Sciences, Complutense University, Madrid, Spain; 7 Pathology Department, Ramón y Cajal Hospital, IRYCIS, Madrid, Spain; 8 Pathology Department, 12 de Octubre Hospital, Madrid, Spain; 9 Medical Oncology Department, 12 de Octubre Hospital, Madrid, Spain; 10 Medical Oncology Department, Ramón y Cajal Hospital, IRYCIS, Madrid, Spain; 11 Faculty of Medicine, Alcalá de Henares University, Madrid, Spain; 12 Faculty of Medicine, Complutense University, Madrid, Spain

**Keywords:** immunotherapy, lung neoplasms, tumor biomarkers, translational medical research, B7-H1 antigen

## Abstract

**Background:**

Tumor mutational burden (TMB) is a recently proposed predictive biomarker for immunotherapy in solid tumors, including non-small cell lung cancer (NSCLC). Available assays for TMB determination differ in horizontal coverage, gene content and algorithms, leading to discrepancies in results, impacting patient selection. A harmonization study of TMB assessment with available assays in a cohort of patients with NSCLC is urgently needed.

**Methods:**

We evaluated the TMB assessment obtained with two marketed next generation sequencing panels: TruSight Oncology 500 (TSO500) and Oncomine Tumor Mutation Load (OTML) versus a reference assay (Foundation One, FO) in 96 NSCLC samples. Additionally, we studied the level of agreement among the three methods with respect to PD-L1 expression in tumors, checked the level of different immune infiltrates versus TMB, and performed an inter-laboratory reproducibility study. Finally, adjusted cut-off values were determined.

**Results:**

Both panels showed strong agreement with FO, with concordance correlation coefficients (CCC) of 0.933 (95% CI 0.908 to 0.959) for TSO500 and 0.881 (95% CI 0.840 to 0.922) for OTML. The corresponding CCCs were 0.951 (TSO500-FO) and 0.919 (OTML-FO) in tumors with <1% of cells expressing PD-L1 (PD-L1<1%; N*=*55), and 0.861 (TSO500-FO) and 0.722 (OTML-FO) in tumors with PD-L1≥1% (N=41). Inter-laboratory reproducibility analyses showed higher reproducibility with TSO500. No significant differences were found in terms of immune infiltration versus TMB. Adjusted cut-off values corresponding to 10 muts/Mb with FO needed to be lowered to 7.847 muts/Mb (TSO500) and 8.380 muts/Mb (OTML) to ensure a sensitivity >88%. With these cut-offs, the positive predictive value was 78.57% (95% CI 67.82 to 89.32) and the negative predictive value was 87.50% (95% CI 77.25 to 97.75) for TSO500, while for OTML they were 73.33% (95% CI 62.14 to 84.52) and 86.11% (95% CI 74.81 to 97.41), respectively.

**Conclusions:**

Both panels exhibited robust analytical performances for TMB assessment, with stronger concordances in patients with negative PD-L1 expression. TSO500 showed a higher inter-laboratory reproducibility. The cut-offs for each assay were lowered to optimal overlap with FO.

## Background

Lung cancer has the leading incidence (11.6% of the cases) and mortality (18.4%) rates of malignant diseases worldwide.^1^ Non-small cell lung cancer (NSCLC) is the most prevalent subtype of lung cancer (85% of cases), and most frequently presents at an advanced stage. Systemic treatments in this setting have evolved, with targeted therapies against specific actionable oncogenic alterations in an increasing list of genes, including *EGFR, ALK* and *ROS1*, among others, now being added to chemotherapy.^2^ However, the emergence of resistances in most patients highlights the urgency for alternative treatment strategies to be developed.

In recent years, the availability of checkpoint inhibitors has had a remarkable impact on outcomes in patients with advanced NSCLC. Treatment with PD-1/PD-L1 inhibitors as single agents or in combination with chemotherapy and/or cytotoxic T-lymphocyte-associated protein 4 (CTLA-4) inhibitors has been particularly successful in pretreated and treatment-naïve patients.^3–8^ However, most of the benefit from these strategies is restricted to a subset of patients displaying long-term survival. At present, patient selection in the clinical practice is based on the immunohistochemical determination of PD-L1 expression by tumor cells, which presents relevant limitations, particularly when checkpoint inhibitors are given in combination.

These limitations on PD-L1 detection are in part due to its expression pattern, influenced by intra-tumoral heterogeneity, temporal and topographical inter-tumoral heterogeneity, impact of previous treatment lines on its expression, the membranous (functional) versus cytoplasmic expression and the type of cell expressing it. In addition, the preanalytical methods and technology used impact on the observed expression due to the instability of the epitopes during fixation and tissue handling and the different affinities and specificities of the available antibodies.^3 9–11^

In this context, there is an urgent need to find optimized and complementary predictive biomarkers for checkpoint blockers.^12^ In recent years, tumor mutational burden (TMB), defined as the total number of mutations per megabase of the tumor genome encoding area, has been identified as a promising predictive tool in many solid tumors,^13–15^ including melanoma^16^ and NSCLC,^17 18^ which present the highest prevalence of somatic mutations.^19^

While whole-exome sequencing (WES) may serve as a more exact method to determine TMB, this technique is nevertheless demanding in terms of coverage, turnaround-time and the required quality and amount of DNA extracted from formalin-fixed, paraffin-embedded (FFPE) tissue. Foundation One (FO) and MSK-Impact^20^ are centralized assays based on next generation sequencing (NGS) panels approved by the US Food and Drug Administration (FDA) for this purpose.^21–24^ Consequently, a range of comprehensive genomic profiling assays (CGPs) has been developed and made commercially available by several companies to facilitate the performing of *in-house* TMB assessments in the clinical practice.^20^

The different CGPs have numerous differences. The most important is the enrichment technology used in each case, but also different preanalytical procedures, methodology workflows, sequencing technologies, recommended depth, horizontal coverage, panel size, gene selection, types of mutations detected, sequencing platforms and bioinformatic algorithms for TMB determination. Several studies have evaluated the major influence that these factors can exert on precise TMB calculations,^20 25–28^ thereby emphasizing the need for standardization studies with the different available approaches. In the present study, we present the results of the “TMB^2^ Harmonization Project: *T*umor *M*utational *B*urden by *T*wo *M*ethods *B*alanced.” We have evaluated the analytical performance of two distributable commercially available panels: TruSight Oncology 500 (TSO500, Illumina) and Oncomine Tumor Mutation Load (OTML, Thermo Fisher) compared with one of the above-mentioned reference methods (FO, Roche). The most important difference is the enrichment method used in each case: TSO500 uses hybrid capture and OTML uses an amplicon-based method. Differences in the methodologies of these two panels include: (i) that TSO500 requires the DNA to be fragmented by sonication previous to library preparation, in order to start with an homogeneous and accurate sample size for the hybrid capture, whereas OTML requires to treat the DNA samples with an uracyl DNA glycosylase enzyme in order to reduce possible deamination events during the amplicon generation; and (ii) that TSO500 uses unique molecular identifiers (UMIs) in the library preparation whereas OTML does not. Ultimately, the main objective of this study is to determine specific TMB cut-off values for each panel to guarantee a correct patient stratification, thus improving the clinical utility of this biomarker.

## Methods

This section can be found in Online supplemental material.

10.1136/jitc-2020-001904.supp1 Supplementary data

## Results

### Analytical performance of the novel NGS panels in TMB determination

**Figure 1:**
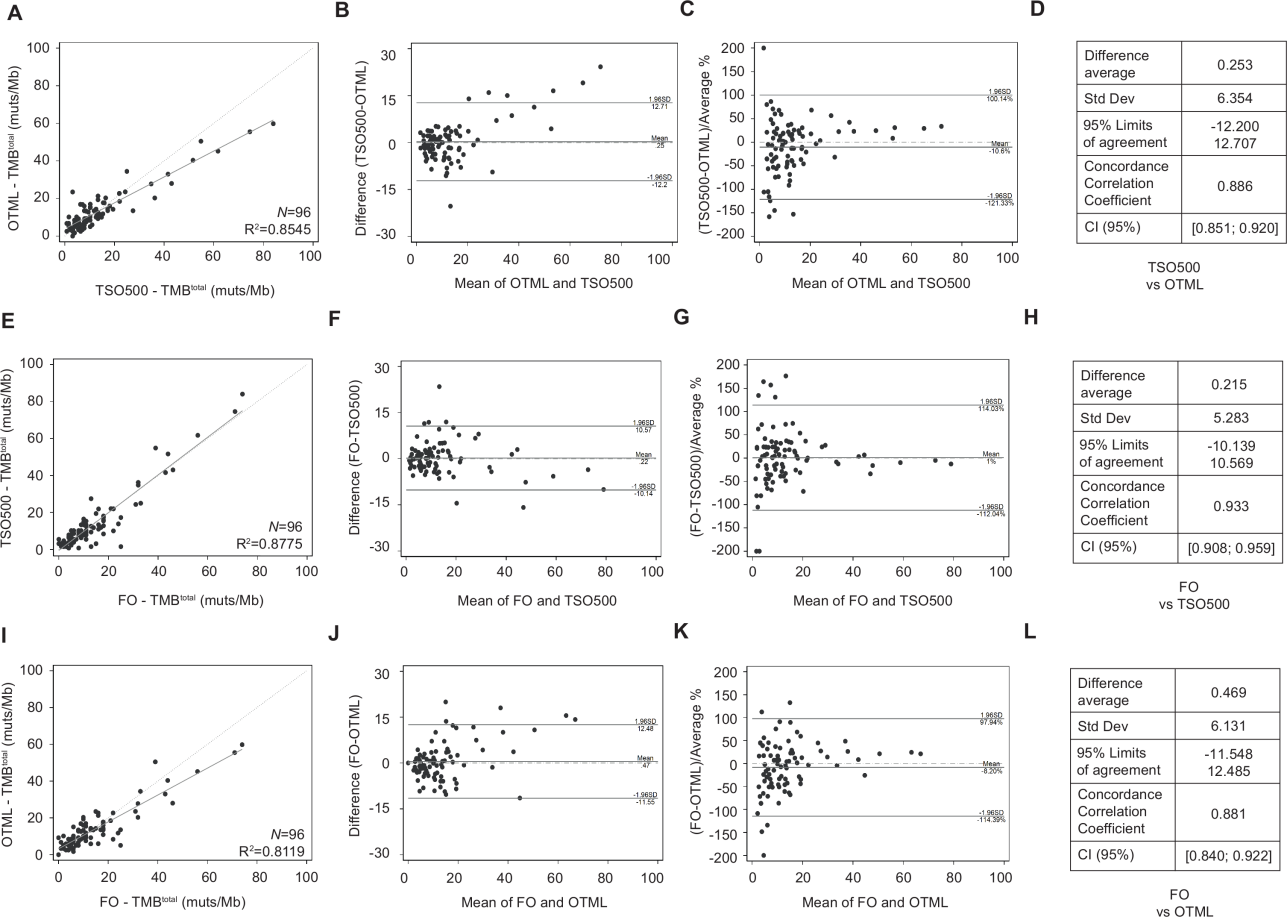
Quantification of the degree of agreement between tumor mutational burden (TMB) determination methods. (A–D) Comparison between TruSight Oncology 500 (TSO500) and Oncomine Tumor Mutation Load (OTML). (E–H) Comparison between Foundation One (FO) and TSO500. (I–L) Comparison between FO and OTML. (A, E, I) Linear regression analyses between methods. (B, F, J) Agreement measurement represented by the difference between the methods against the average of both methods (Bland-Altman plots). (C, G, K) Agreement measurement represented by the difference between the methods divided by the average %, versus the mean of both methods. Transformation of Bland-Altman plots with a correction that allows for easier visualization. (D, H, L) Degree of agreement shown by the average difference, SD, 95% limits of agreement, concordance correlation coefficient and 95% CI. TMB was calculated as total (synonymous and non-synonymous mutations per megabase of DNA) in a cohort of N=96 early-stage non-small cell lung cancer tumors.

A set of 96 resected early-stage NSCLC tumors in FFPE format was used to assess the analytical performance of the TSO500 and the OTML panels. The median total TMB (TMB^total^, synonymous and non-synonymous mutations) calculated by the TSO500 panel was 8.8 mutations/Mb, spanning a range from 0.8 to 84 muts/Mb. In contrast, the median TMB^total^ calculated by the OTML panel was 9.7 muts/Mb, ranging from 0 to 59.8 muts/Mb. A linear regression analysis of TMB^total^ calculated with OTML versus TSO500 is shown in figure 1A, where R^2^=0.8545. A Bland-Altman plot describing agreement between the two analytical methods is shown in figure 1B. The systematic difference in absolute terms was close to zero (0.253 (−12.200; 12.707)), with seven measurements (7.29%) outside of the 95% limits of confidence for agreement between OTML and TSO500, showing higher values for TSO500. In general, the mean values (horizontal axis) between each pair of measurements showed higher differences (vertical axis). These differences were corrected when data were plotted according to relative values (figure 1C). The concordance correlation coefficients (CCC) for TSO500 versus OTML was 0.886 (figure 1D) (95% CI 0.851 to 0.920).

### Analytical performance of the NGS panels compared with the reference standard method

Total TMB values obtained with FO in the 96 sample set had a median value of 10 muts/Mb, spanning a range of TMB values from 0 muts/Mb to 74 muts/Mb. Linear regression analyses of TMB values calculated with TSO500 versus those obtained by FO resulted in a R^2^=0.8775 (figure 1E), while that for OTML versus FO was R^2^=0.8119 (figure 1I). The level of agreement between each pair of methods is shown in figure 1F, G, J, K. The systematic differences were close to zero (0.215 (−10.139; 10.569)), with six measurements (6.25%) outside of the 95% limits of confidence for agreement between FO and TSO500, and 0.469 (−11.548; 12.485) with five measurements (5.21%) outside of the 95% limits of confidence for agreement between FO and OTML. CCCs were 0.933 (95% CI 0.908 to 0.959) for FO versus TSO500, and 0.881 (95% CI 0.840 to 0.922) for FO versus OTML (figure 1H, L). A summary of all TMB results with the three panels is provided in online supplemental table S1.

10.1136/jitc-2020-001904.supp2 Supplementary data

### TMB concordance within tests in patients grouped according to PD-L1 expression

**Figure 2:**
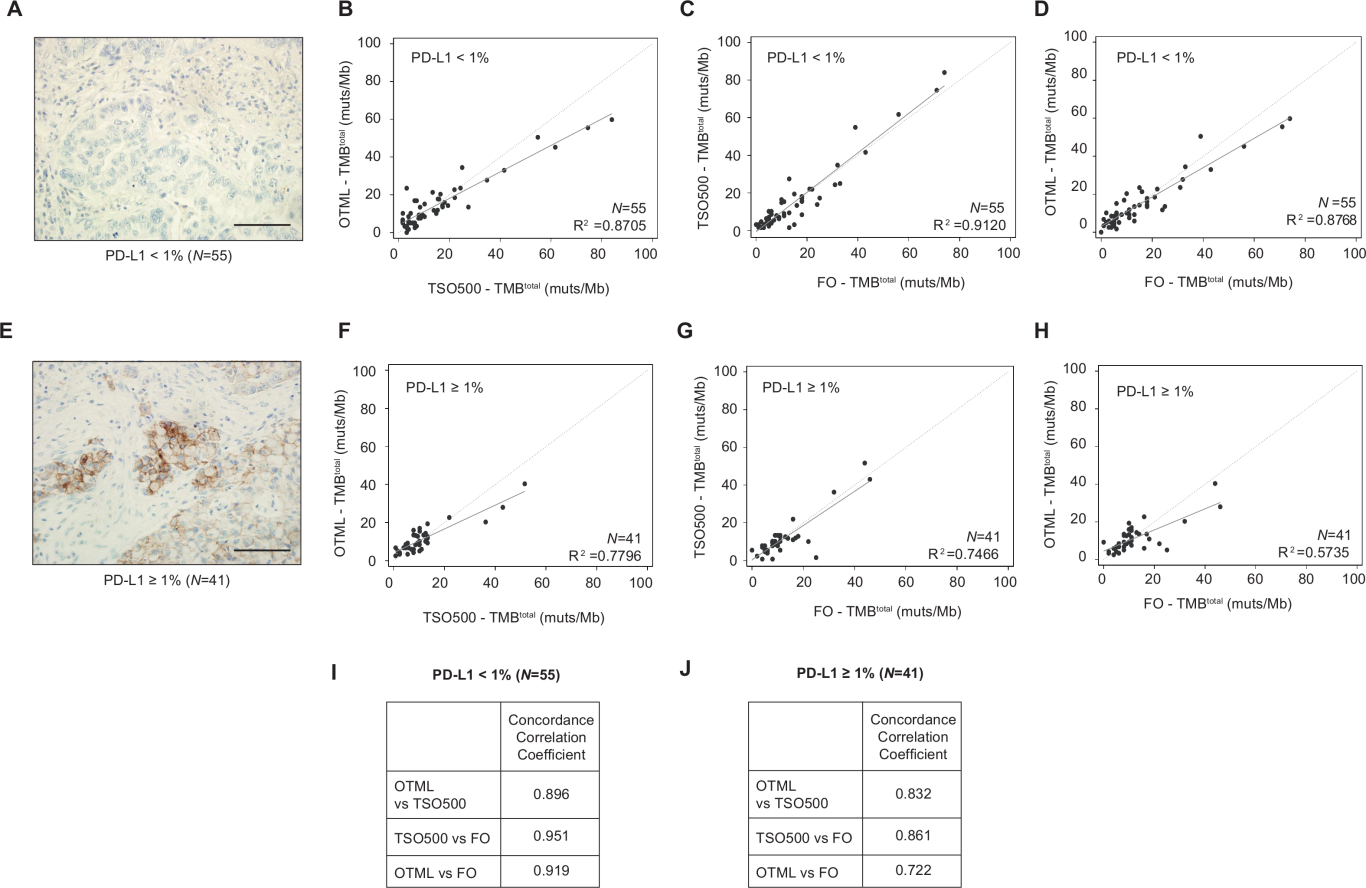
Quantification of the degree of agreement between tumor mutational burden (TMB) determination methods in non-small cell lung cancer (NSCLC) samples categorized by PD-L1 expression. (A, E) Images of NSCLC tumor samples categorized by their PD-L1 expression, as detected by immunohistochemistry with anti-PD-L1 22C3 PharmDx antibody. Tumor samples were categorized as: PD-L1<1% (A, N=55) or PD-L1≥1% (E, N=41). (B–D, F–H) Correlation plots of the TMB^total^ of Oncomine Tumor Mutation Load (OTML) versus TruSight Oncology 500 (TSO500) (B, F), TSO500 versus Foundation One (FO) (C, G) and OTML versus FO (D, H) for PD-L1-negative and PD-L1-positive groups, respectively, as indicated in the graphs. (I, J) Degree of agreement shown by the concordance correlation coefficients based on three comparisons for each group of tumors. Bland-Altman plots and other parameters such as average difference, SD, 95% limits of agreement, and 95% CI of the CCCs are shown in online supplemental figures 3 and 4. Scale bar represents 100 µm.

We analyzed the extent of agreement among the tests in subgroups of NSCLC samples separated by PD-L1 expression. Percentages of cells expressing PD-L1 were analyzed in patient samples and classified as PD-L1<1% (N=55) or PD-L1≥1% (N=41). Correlation analyses of tumors with PD-L1 expression<1% (N=55) provided R^2^ values of 0.9120 for TSO500 versus FO and 0.8768 for OTML versus FO (figure 2A–D). In the group expressing PD-L1≥1% (N=41), R^2^ values of 0.7466 for TSO500 versus FO and 0.5735 for OTML versus FO (figure 2E–H) were obtained. CCC values of 0.951 (95% CI 0.926 to 0.975) for TSO500 versus FO and 0.919 (95% CI 0.882 to 0.956) for OTML versus FO (figure 2I and online supplemental figure S3) were consistent with the correlation analyses in PD-L1<1% tumors. In contrast, in samples expressing PD-L1≥1%, a CCC value of 0.861 (95% CI 0.781 to 0.942) for TSO500 versus FO was determined compared with 0.722 (95% CI 0.586 to 0.858) for OTML versus FO (figure 2J and online supplemental figure S4). Data for the graphs shown and statistical analyses of the differences in the demographic data between the two groups of patients are presented in online supplemental table S2.

10.1136/jitc-2020-001904.supp3 Supplementary data

### Reproducibility analysis: inter-laboratory cross-validation studies

**Figure 3:**
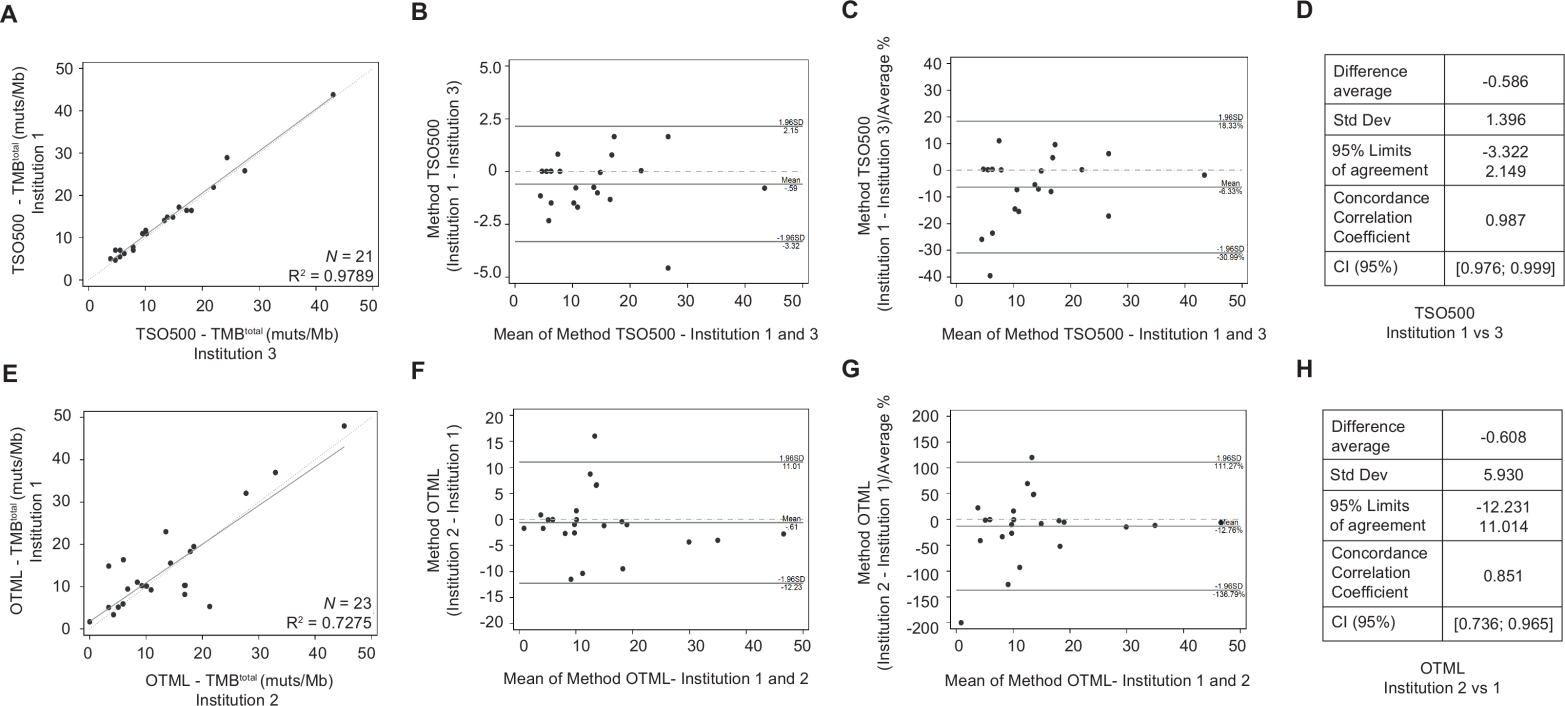
Inter-laboratory reproducibility tests for TruSight Oncology 500 (TSO500) and Oncomine Tumor Mutation Load (OTML) assays. Inter-laboratory cross-validation analyses were performed on a proportion ~25% of the sample cohort. Each test used the same set of input DNAs in parallel, was assessed by different operators, and in two distinct hospitals. (A–D) TSO500 was tested in parallel on the same 21 DNA samples in Hospital Ramón y Cajal (Institution 3) and Hospital 12 de Octubre (Institution 1). (E–H) OTML was assessed in parallel on the same 23 DNA samples in Hospital 12 de Octubre (Institution 1) and Hospital HM Sanchinarro (Institution 2). (A, E) Linear regression plots for each of the tests: TSO500 (A) and OTML (E). (B, F) Bland-Altman plots showing differences between the methods versus the average of both methods. (C, G) Bland-Altman plots showing differences between the methods divided by the average %, versus the average of both methods. (D, H) Degree of agreement shown by the average difference, SD, 95% limits of agreement, concordance correlation coefficient and 95% CI. TMB, tumor mutational burden.

To evaluate the inter-laboratory reproducibility of each NGS panel, a subset of samples was analyzed using the same test in two different laboratories. Results are shown in figure 3 and online supplemental table S3. The concordance was very good for both panels. More robust data were obtained for TSO500, with a CCC of 0.987 (95% CI 0.976 to 0.999) and R^2^=0.9789, whereas for OTML the CCC was 0.851 (95% CI 0.736 to 0.965) and R^2^=0.7275. Of note, the limits of agreement at 95% were much narrower for TSO500 (−3.322 to 2.149) than for OTML (−12.231 to 11.014).

10.1136/jitc-2020-001904.supp4 Supplementary data

### Performance of the NGS panels selecting TMB^high^ tumors

**Figure 4:**
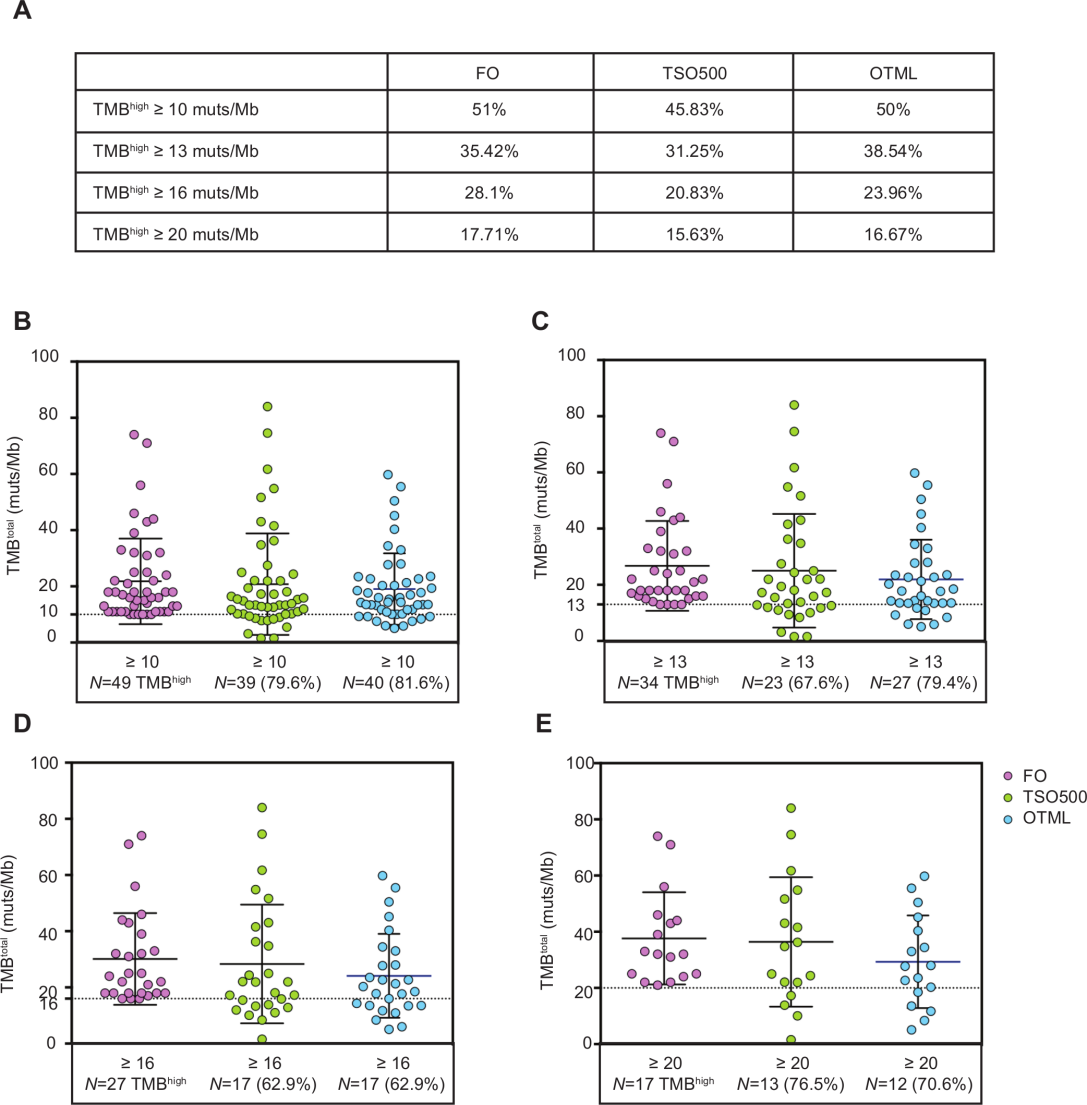
Visual distribution of tumor mutational burden (TMB) values obtained with TruSight Oncology 500 (TSO500) or Oncomine Tumor Mutation Load (OTML) for samples categorized as TMB^high^ with Foundation One (FO), using different cut-off values. (A) Summary of the percentage of patients from the study cohort (N=96) that would be categorized as TMB^high^ using four different cut-off values for the two panels versus FO. Care should be exercised interpreting the data, as the percentages are similar; patients included in the high or med/low categories are interchangeable between the tests if the same cut-offs are used. (B–E) Samples were selected in the TMB^high^ category based on their total TMB value obtained with FO test and selected according to the four different cut-offs: TMB^total^ ≥10 (B, N=49), ≥13 (C, N=34), ≥16 (D, N=27) or ≥20 (E, N=17) muts/Mb, respectively. Only samples above the selected threshold by FO (samples in pink) are likewise plotted in the same graph for TSO500 and OTML. TMB values obtained with TSO500 (green) and OTML (blue) are shown. Several patients that are categorized as TMB^high^ according to the standard method would have been missed with TSO500 and/or OTML if the same cut-off value was maintained. Some adjustments were necessary to increase the sensitivity of the tests.

We analyzed four different cut-off values to define TMB status (high vs low) with FO: 10, 13, 16 and 20 muts/Mb. We focused in those four cut-off values as they were previously used as predictive in different clinical trials (10 muts/Mb in Checkmate 227^7^; 13 muts/Mb in Checkmate 026^29^; 16 and 20 muts/Mb in the Mystic Trial^30^). When using a cut-off value of 10 muts/Mb, FO categorized 51% of patients from the cohort as TMB^high^ (≥10 muts/Mb), whereas TSO500 and OTML assigned the same status to 45.83% and 50% of patients, respectively (figure 4A). Among patients identified as TMB^high^ by FO, 20.40% and 18.40% scored as TMB^low^ (<10 muts/Mb) with the TSO500 and OTML assays, respectively (figure 4B). In addition, out of the 47 tumors with TMB^low^, 10.60% and 17% scored as TMB^high^ in the TSO500 and OTML assays, respectively (online supplemental figure S5). If a higher cut-off point is desired, such as 13, 16 or 20 muts/Mb, the percentage of patients classified as TMB^high^ with panel TSO500 or panel OTML would be lower than the percentage of patients that would be categorized as TMB^high^ by FO (using those same cut-off values; figure 4C–E). Consistently, the percentage of patients categorized as TMB^low^ would be higher with TSO500 or OTML than with FO (online supplemental figure S5).

### Selection of adjusted cut-off points for each NGS panel

**Figure 5:**
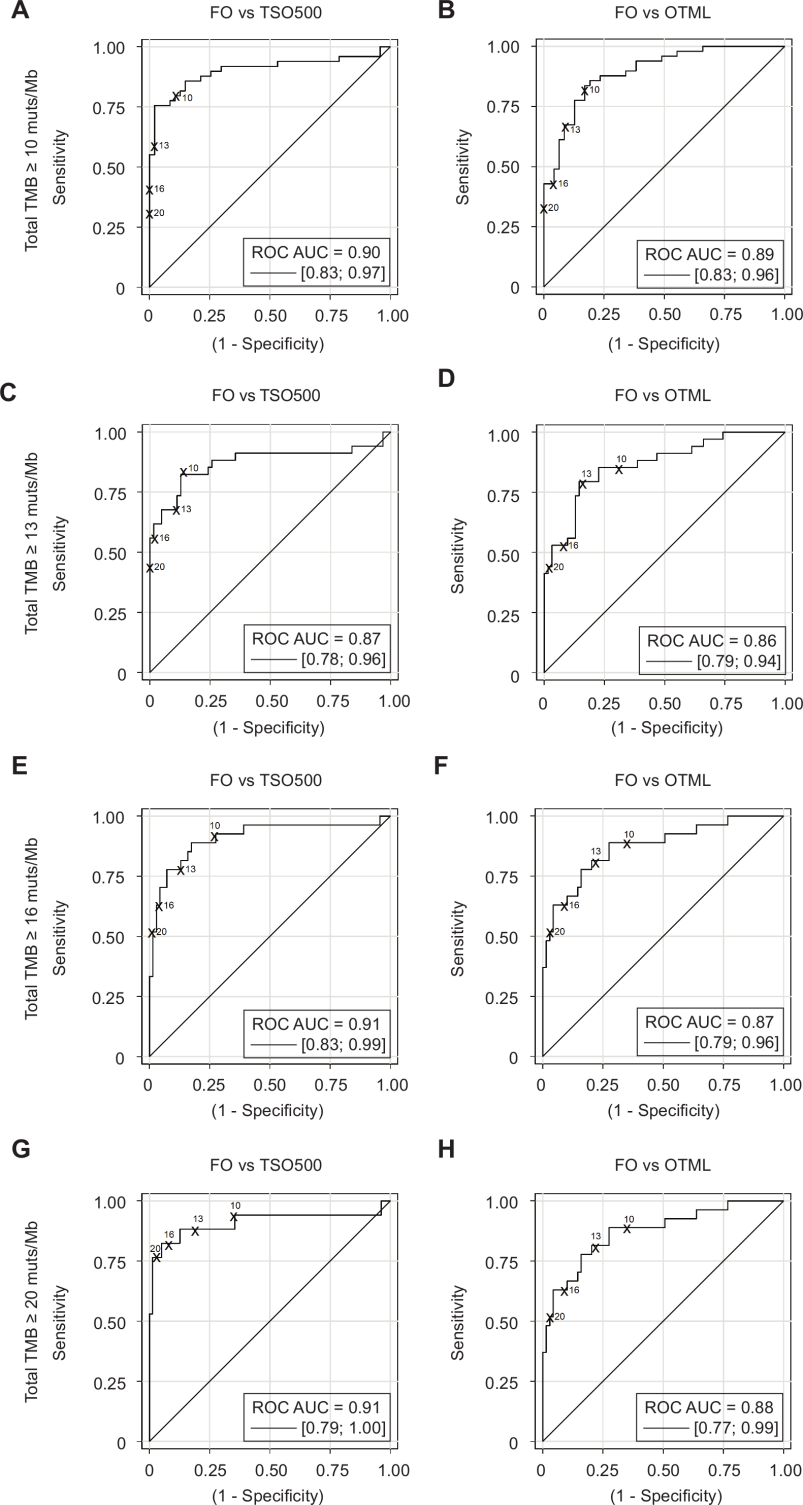
Evaluation of the sensitivity, specificity, positive predictive value and negative predictive value of the tests. Receiver operating characteristic (ROC) curves for the TruSight Oncology 500 (TSO500) versus Foundation One (FO) (A, C, E, G) and Oncomine Tumor Mutation Load (OTML) versus FO (B, D, F, H) comparisons are shown. Graphs were plotted by selecting four different high tumor mutational burden (TMB^high^) cut-off values for the FO test: TMB^total^ ≥10 muts/Mb (A, B, N=49), ≥13 muts/Mb (C, D, N=34), ≥16 muts/Mb (E, F, N=27) or ≥20 muts/Mb (G, H, n=17). Assuming that FO is the reference test, values above the selected cut-off in each case are positives and values under the selected cut-off are negatives. The behavior of the panel under evaluation is then observed in a continuous model. Areas under the curve (AUC) are shown for each graph. The four theoretical cut-off values are indicated in the eight different graphs. Empirical cut-off values have been statistically obtained, as well as the adjusted cut-off values for each panel that are proposed to be equivalent to the four different cut-off values for FO. Complete datasets are available in online supplemental table S4.

To determine the adjusted cut-offs for TSO500 and OTML corresponding to TMBs ≥10, 13, 16 and 20 with FO, characteristics of the area under the receiver operating characteristic (ROC) curve were calculated (figure 5). The discriminatory capacities of TSO500 and OTML tests with respect to a FO value of TMB ≥10 were 0.90 (95% CI 0.83 to 0.97; figure 5A) and 0.89 (95% CI 0.83 to 0.96; figure 5B), respectively (p value=0.8398). Similarly, no significant differences between the discriminatory capacities of TSO500 and OTML with respect to FO were found for TMB≥13 (p value=0.8625), TMB≥16 (p value=0.2284) or TMB≥20 (p value=0.2308) (figure 5C–H and online supplemental table S4).

10.1136/jitc-2020-001904.supp5 Supplementary data

Table 1 shows the adjusted cut-off values calculated for both TSO500 and OTML, corresponding to cut-off points of 10, 13, 16 and 20 muts/Mb for the FO assay. These values represent a compromise between maximum sensitivity (>88%) and specificity obtained from the ROC curves. A comprehensive list of continuous values for both tests, analyzed in relation to each of the desired cut-off points by FO (10, 13, 16 and 20 muts/Mb), including sensitivity, specificity, positive predictive value (PPV) and negative predictive value (NPV) for each point are shown in online supplemental table S4. Cut-off points that guarantee a sensitivity >88% corresponding to 10 muts/Mb by FO would be 7.847 (for TSO500) and 8.380 (for OTML), corresponding to 13 muts/Mb by FO would be 9.434 (for TSO500) and 9.240 (for OTML), respectively, corresponding to 16 muts/Mb by FO would be 10.995 and 10.90 and for 20 muts/Mb by FO would be 13.842 and 11.73, respectively.

**Table 1:** Adjusted cut-offs calculated for TSO500 and OTML tests, equivalent to different cut-off points for the FO reference standard

Reference variable	FO≥10 muts/Mb		FO≥13 muts/Mb		FO≥16 muts/Mb		FO≥20 muts/Mb	
Classification variable	TSO500	OTML	TSO500	OTML	TSO500	OTML	TSO500	OTML
Adjusted cut-off point with sensitivity >88%	7.847	8.380	9.434	9.240	10.995	10.900	13.842	11.730
Sensitivity at cut-off point (%)	89.90	89.90	88.24	88.24	88.89	88.89	88.24	88.24
Specificity at cut-off point (%)	74.47	65.96	74.19	61.29	82.61	72.46	87.34	65.82
Positive predictive value at cut-off point (%)	78.57	73.33	65.22	55.56	66.67	55.81	60.00	35.71
Negative predictive value at cut-off point (%)	87.50	86.11	92.00	90.48	95.00	94.34	97.18	96.30

Cut-off points of 10, 13, 16 and 20 muts/Mb were selected for FO, and the performance of the two next generation sequencing tests was evaluated by continuously interrogating the sensitivity and specificity at each point, as well as positive and negative predictive values, to discern high tumor mutational burden versus med/low tumors compared with FO. We selected as adjusted the points that offer maximum sensitivities (>88%) and negative predictive values for each test and for each equivalent cut-off value for the FO test. Adjusted cut-off values were lower than those for FO. The complete set of values is shown in online supplemental table S4.

FO, Foundation One; OTML, Oncomine Tumor Mutation Load; TSO500, TruSight Oncology 500.

### Degree of immune infiltration in tumors with different TMBs

At last, we wanted to analyze the presence of different immune infiltrates in the tumors with respect to their mutation burden. For that, we stained four additional slides of each of the 96 tumors with markers for four different populations of immune infiltrates: CD4^+^ T cells, CD8^+^ T cells, CD20^+^ B cells and CD68^+^ macrophage/monocytic populations. We did not find significant differences that indicated a correlation between higher TMB and higher or lower immune infiltration of these four cell categories. However, we found a trend indicating that CD4^+^ T cells tend to be more infiltrated in the tumors with lower TMB, and CD68^+^ macrophage/monocytic populations tend to concentrate in tumors with higher TMB. The results are shown in online supplemental figure S6 and table S5.

10.1136/jitc-2020-001904.supp6 Supplementary data

## Discussion

The calculation of TMB as a guiding biomarker for treatment with checkpoint inhibitors in clinical practice largely lies in the implementation of NGS gene panels. In this work, we used a cohort of 96 NSCLC samples to determine the technical performance of TSO500 and OTML assays in comparison to a reference standard method (FO). We verified an adequate degree of agreement among the methods, with concordance values tending to be higher for tumors expressing lower levels of PD-L1. We provided a range of cut-off values for both TSO500 and OTML corresponding to different levels of TMB that have been of interest in previous immuno-oncology trials in NSCLC, and estimated their sensitivities, specificities, PPVs and NPVs.

Immunotherapy with checkpoint inhibitors has impacted treatment outcomes in many solid tumors, including lung cancer.^4 31 32^ However, up to 60%–80% of patients with advanced NSCLC will not benefit to any significant extent from PD-1/PD-L1 inhibitors.^5 31 33 34^ The only clinically validated biomarker in this context is the tumor expression of PD-L1, as measured by immunohistochemistry, and its predictive capacity is far from optimal, particularly when PD-1/PD-L1 inhibitors are combined with chemotherapy, radiotherapy or CTLA-4 inhibitors.^6 7 33 35–37^ The role of TMB in this context is unclear, as the survival benefit with ipilimumab plus nivolumab over chemotherapy in the Checkmate 227 trial—the only trial performed in this setting that had patients selected for a primary end-point on the basis of tumor TMB (≥10/Mb)—was independent of the TMB scores, even though TMB was predictive of objective response rate and progression free survival.^37^ Relevantly, TMB may also be predictive of overall survival benefit for high PD-L1 expression patients when treated with pembrolizumab as single agent.^38^ However, TMB may not be predictive of survival when pembrolizumab is administered in combination with chemotherapy.^39^ Other studies demonstrated that using higher TMB values, the selection of patients was more accurate in predicting the greatest benefit from therapy.^30^ Of note, recently the US FDA approved pembrolizumab for the treatment of adult and pediatric patients with unresectable or metastatic solid tumors with tissue TMB-high (TMB-H; ≥10 mutations/megabase), as determined by an FDA-approved test, who have progressed following prior treatment and have no satisfactory alternative treatment options.^40^

Calculation of the TMB by WES is highly time-consuming, making it less feasible to guarantee an appropriate turnaround time in the clinical practice. Several NGS panels with predictive capacity in the clinic are currently approved by the FDA for the determination of TMB: FO, F1CDx, MSK Impact, Omics Core and PGDx elio tissue complete.^22–24 41 42^ Recently, several biotech companies specializing in NGS have released their own panels for TMB determination from tumor tissue samples.^20^ Their agreement with the gold standard method (WES) or with approved large panels able to estimate the TMB -such as FO- are currently being independently analyzed in large cohorts of clinical samples.^43^ This is one of the first harmonization studies of TMB determination, in which we evaluated 96 clinical NSCLC samples. There was a high correlation among the overall TMB values obtained with the three different NGS panels analyzed (FO-TSO500 R^2^=0.8775; FO-OTML R^2^=0.8119; TSO500-OTML R^2^=0.8545). More importantly, the actual values obtained with each method were highly concordant as reflected by their CCCs (ie, there was a high correlation among the overall TMB values obtained with the three different NGS panels analyzed (FO-TSO500 CCC=0.933; FO-OTML CCC=0.881; TSO500-OTML CCC=0.886)). We have noticed a few deviations such as a small percentage of samples that show higher values of TMB with TSO500 than with OTML (figure 1B, 5% of samples outside of the limits of confidence). Those six samples have specific genes frequently mutated that, in a high percentage (52%), are not included in the design of the OTML panel, but they are included in the design of the TSO500. That can slightly change the total TMB number calculated with one panel versus another. These results are providing numeric proof that gene content is as important as horizontal coverage in the design of NGS panels for TMB determination, a concept in continuous open discussion in the field. This is the main reason why cut-offs need to be optimized and adjusted for each different panel.

The need for standardization and harmonization of TMB assessments across the available methodologies is obvious owing to their inherent differences in megabases of exonic coverage, selection of genes under study (some genes have hotspots that are more prone to mutate than others, and each panel has a different gene content), amplification and library generation technologies, presence or absence of UMIs, sequencing technology, and TMB calculation algorithm (including the method of correction for deaminations caused by formalin fixation, as well as the reading depth), among others. Countless efforts have been made to address this common goal.^27 44^ Some studies approached the TMB harmonization from an *in silico* perspective^45^ or with relatively reduced cohorts,^46^ while other studies have overcome the cohort size difficulties but still lack certain critical evaluations such as the definition of specific TMB cut-offs equivalent to those established with the reference method^47^ or inter-laboratory reproducibility assays.^43^ Apart from the present study, two other broad studies of TMB standardization have been carried out: one by Friends of Cancer Research and other by Quality in Pathology (QuIP) study investigators. In the first case, a first phase of the study analyzed data from The Cancer Genome Atlas with WES and 11 simulated NGS panels in order to create a calibration and validation set for TMB calculation. The performance of this validation set was evaluated in a second phase of the study with 25 FFPE samples.^48 49^ In the QuIP initiative, 20 FFPE samples were evaluated in 15 participating institutions, each of which assessed six panels. Specific TMB cut-offs were defined corresponding to 199 mutations determined by WES, and equivalent to 10 mutations per megabase as determined by FO.^50^ For most of the panels evaluated, including TSO500, the calculated TMB cut-offs were in the range of 9.4 to 11.5 muts/Mb. Among the exceptions, OTML cut-offs were 7.8–7.9, depending on the regression methodology used.

It was relevant to us to evaluate that the performance of the tests was still robust in the population of PD-L1 positives, compared to the general population. That is the specific population of patients with NSCLC for which the FDA has approved the use of pembrolizumab (anti-PD-1). Indeed, exploratory analysis of the Keynote 042 trial suggests that among patients with tumors expressing PD-L1 in ≥50% of cells, only those whose TMB was higher than the median exhibited any therapeutic benefit with PD-1/PD-L1 inhibitors as compared to chemotherapy.^38^ We consider particularly relevant to analyze the agreement of the three methods in the two separated subgroups, because the PD-L1 positive patients are the ones in which the TMB evaluation will have putative clinical utility. The results in our cohort of 96 patients seem to indicate that the higher the PD-L1 the lower the TMB. Moreover, linear regression values were stronger in the PD-L1-negative tumors, with a N of 55 samples, than in PD-L1-positive tumors, with a N of 41 samples.

One of the best attributes of TMB as a biomarker is its ease of quantification on a continuous scale. However, biomarkers in the clinic are frequently used in a binary fashion (eg, high or low). When this was done with TMB, regardless of the cut-off point used to define high and low, we found that a significant number of patients (around one third) are misclassified as compared to the reference test using the same threshold. In fact, when we analyzed the ROC curves, it could be appreciated that for any cut-off with the FO panel, the corresponding cut-off point with the TSO500 panel needed to be lowered, and even more so with the OTML panel. We have thus obtained here a set of empirical, adjusted cut-off values—representing reasonable compromises between acceptable sensitivity and specificity—for a variety of different cut-off points from the FO panel that have been pursued in different clinical scenarios. Indeed, the cut-point of TMB for patient selection might depend on the treatment context including line of therapy (first line or salvage treatment), specific therapy (single agent immunotherapy or in combination with chemotherapy or radiotherapy), stage of disease (adjuvant setting, stage III or metastatic disease) and further research in this area is warranted. We have selected in this study four relevant cut-off values in lung cancer that were previously used as predictive in different clinical trials: 10 muts/Mb (Checkmate 227^7^); 13 muts/Mb (Checkmate 026^29^); 16 and 20 muts/Mb (Mystic Trial^30^). We have also provided cut-off values for the novel tests that would increase sensitivity (reducing the false negative rate) in relation to the FO test, always taking into account the concurrent effect on increasing the false positive rate. However, it must be mentioned that other small studies obtained different cut-off values for TSO500 when compared with FO.^43^ Efforts to standardize the cut-off for a TMB high population across platforms may be influenced by several factors, including cohort specific effects, preanalytical differences in tissue processing, and the statistical approaches utilized to select the appropriate threshold, which may lead to differences across studies.

We were also interested here in determining the reproducibility of each test when used in different hospital laboratories by different operators. When around one quarter of the sample cohort was studied with TSO500 and OTML in two paired institutions, the outcomes for both assays were highly reproducible. To this end, TSO500 was found to be more robust, with a R^2^ of 0.9789 and a CCC of 0.987 (N=21) compared with a R^2^ of 0.7275 and a CCC of 0.851 for OTML (N=23). The TSO500 panel is a hybrid capture panel, not amplicon-based, thereby resulting in a lower number of artifacts which may account for its better reproducibility. TSO500 uses UMIs, which avoids artifacts caused by errors in the polymerase and also by incorrect interpretation of deamination, because each of the original DNA strands are specifically labeled with a barcode that distinguishes original mutations from mutations that appear during the library preparation. Furthermore, the pipeline analysis included an automatic algorithm that corrects for deaminations occurring as a consequence of the formalin fixation of the tissue in the case of the TSO500 assay. In contrast, when using the OTML assay, the operator must manually correct cases of high deamination as per the manufacturer’s guidelines. Therefore, an obvious conclusion is that, in order to use the OTML panel for TMB determination across laboratories, it is essential to standardize the processing criteria when testing samples with high deaminations.

At last, we evaluated the degree of infiltration of different immune populations (CD8^+^ T cells, CD4^+^ T cells, B cells and macrophages) with respect to the TMB of each tumor. We observed that none of the four populations seem to correlate with the TMB with statistical significance, although the infiltration with CD4^+^ T cells shows a trend of higher accumulation in tumors with lower TMB and less presence in tumors with higher TMB, and CD68^+^ macrophages seem to present the opposite trend where more macrophages are infiltrated in tumors with higher TMB. It is feasible to hypothesize that, in the case of macrophages, they would be called to the tumors that harbor more mutations and resultant antigens. In the case of the CD4^+^ T cells, it would depend on the specific cell subtypes of cells that are being recruited to the tumor. Therefore, further investigation regarding the determination of specific cell subtypes (regulatory vs active CD4^+^ T cells, M1 vs M2 macrophages) will be needed.

In conclusion, our work presents a comprehensive harmonization study for TMB determination using the TSO500 and OTML panels versus the FO reference standard method. Our results obtained on FFPE tumor tissues from a cohort of 96 patients are empirical and not *in silico*. The results are satisfactory, and both tests on the FFPE samples performed very well both analytically and clinically. Bland-Altman analyses demonstrated good concordance between both tests and the FO reference standard. For the specific characteristics of our study, we described in detail that in order to appropriately select TMB^high^ patients and not overlook any putative candidates for therapy, the cut-offs needed to be lowered compared to those selected for FO. The fact that cut-offs in FO resulted higher than for TSO500 and OTML could indicate that the selection of genes that are included in FO are more prone to be mutated than the selection of genes included in the other two panels. Or that the algorithm that FO introduces to discriminate deamination artifacts due to formalin fixation of the samples is better optimized. Moreover, we determined the adjusted cut-offs for different sensitivities and specificities, equivalent to four different FO cut-off points: 10, 13, 16 and 20 muts/Mb. The results should thus serve as a highly valuable tool for laboratories and hospitals to select appropriate cut-offs when using these two panels, providing information about different empirical values of specificity and sensitivity, and thus facilitating patient selection. Moreover, we have studied the reproducibility of both tests from an inter-laboratory perspective, and the behavior of the tests in two different groups of tumors based on their different PD-L1 expression levels. These findings should have an immediate impact on clinical practice and in the personalized management of patients with lung cancer.

10.1136/jitc-2020-001904.supp7 Supplementary data

10.1136/jitc-2020-001904.supp8 Supplementary data

10.1136/jitc-2020-001904.supp9 Supplementary data

## Data Availability

All data relevant to the study are included in the article or uploaded as supplemental information.
