# Supplementary material for: Tumor mutational burden assessment in non-small-cell lung cancer samples: results from the TMB2 harmonization project comparing three NGS panels
Source: J Immunother Cancer. 2021 May 7;9(5):e001904. doi: 10.1136/jitc-2020-001904 (PMC8108670; doi:10.1136/jitc-2020-001904)

Online Supplemental Material

SUPPLEMENTAL METHODS

NSCLC samples and patient cohort

A cohort of 110 resected early stage NSCLC tumors was gathered. After resection, tumor samples were formalin-fixed and paraffin-embedded (FFPE) and stored in the Pathology Department of 12 de Octubre University Hospital, under signed agreed consent. Protocol was approved by the Ethics Committee of the Hospital 12 de Octubre with identification number #18/385. However, out of the 110 samples, only 96 fulfilled the quality requirements for the three panels (FO, TSO500 and OTML). A flowchart of the study is shown in the **Supplemental Figure S1**. Clinical and demographic information of the patient cohort can be found in the **Supplemental Table S6**, including a comparison of the demographic characteristics between the TMB^{high} and $TMB^{med/low}$ groups of patients, as determined by FO using a cutoff of 10 mutations/Megabase.

DNA extraction, quantification and quality measurement

For every sample of the cohort (N=110), 10 sections of 4 µm of FFPE tissue were subjected to DNA extraction. FFPE tissue deparaffination was performed with mineral oil. DNA extraction was carried out with the QIAmp DNA FFPE Tissue kit (#56404, QIAgen). Quantification was performed by fluorometry with the Qubit dsDNA HS (High Sensitivity) Assay Kit (#Q32854, Invitrogen). After DNA extraction and quantification, DNA quality was evaluated using the FFPE QC kit (#15013664, Illumina) by comparing the amplification efficiency versus a fresh control tissue provided by the manufacturer. The IQ SYBR Green Supermix (#170-8880, BioRad) was used to perform the

Ramos-Paradas J, et al. J Immunother Cancer 2021; 9:e001904. doi: 10.1136/jitc-2020-001904

amplification reaction. All DNA extractions, quantifications and quality evaluations were performed at 12 de Octubre University Hospital by the same person in order to avoid bias. Afterwards, DNA samples were shipped to HM Sanchinarro for Oncomine TML library preparation and sequencing, as well as to Hospital Ramón y Cajal for inter-laboratory cross-validations. Characteristics of the NGS panels selected for the study are found in **Supplemental Table S7**. Information regarding DNA concentration and quality can be found in **Supplemental Table S8.**

TMB assessment with the Reference Standard Method

Comprehensive genomic profiling was performed using the Foundation One Assay and the Foundation One CDx Assay (Cambridge, MA) as the reference standard method in our harmonization study. Foundation One CDx has been clinically validated (based on demonstrated concordance with cobas® EGFR mutation test, Ventana ALK (D5F3) CDx Assay, Vysis ALK Break-Apart FISH Probe Kit, therascreen® KRAS RGQ PCR Kit, Dako HER2 FISH PharmDx® Kit, cobas® BRAF V600 Mutation Test, THxID® BRAF kit) and analytically validated – with 9x more samples and cell lines validated for FoundationOne CDx as compared with FoundationOne, which is analytically validated[1, 2]. From now on, we will refer to these two assays as Foundation One (FO). For the whole cohort (N=110), 14 slides of 4 µm from FFPE blocks were sent to Roche FMI (Penzberg, Germany) through its Spanish distributor Roche Pharma S.A, to perform the Foundation One Assay. For each tumor, an hematoxilin-eosin stained slide was used to determine histology, tumor cellularity and pathologic diagnosis. DNA extraction was only performed in samples with >20% tumor cellularity. The average tumor cellularity of our cohort was 74% (**Supplemental Table S8)**. For library generation, hybridization-capture of exonic regions from 315 cancer-related genes and selected introns from 36

genes commonly rearranged in cancer (with a total number of studied genes of 324) was applied to 50-200 ng of DNA extracted from FFPE clinical cancer specimens. Libraries were sequenced to high, uniform median coverage (>500X) and assessed for base substitutions, short insertions and deletions, copy number alterations, gene fusions/rearrangements and ultimately, determination of total Tumor Mutational Burden. From the 110 samples sent to Foundation One Medicine Inc., 6 did not meet the quality requirements of the test and Tumor Mutational Burden could not be determined. Therefore, the Foundation One Assay was performed successfully for *N*=104 samples. Half of the evaluated samples were described with the Foundation One Assay and the other half was described with the Foundation One CDx Assay. An analytical comparison between FO and F1CDx was performed to guarantee that the Tumor Mutational Burden was properly determined independently of the assay version. This comparison is shown in **Supplemental Figure S2**.

In-house NGS libraries preparation and sequencing

Illumina TruSight Oncology 500 (TSO500). TSO500 was performed under an Early Access Agreement. 104 patients were sequenced with TSO500. Four samples with an unsuccessful report with FO had already been described with the TSO500 and were excluded of subsequent analyses. Therefore, the total number of valid samples described with TSO500 was 100. TSO500 targets 523 cancer-relevant genes with a coverage of 1,94 Mb, surpassing requirements to calculate TMB[3]. Libraries were prepared following the manufacturer instructions. Briefly, DNA was sheared with a M-220 Ultrasonicator (Covaris). Total DNA input used was 40/60 ng in a total volume of 52 µl (DNA fragmentation concentration = 0,76/1,15 ng/µl, respectively). Samples were fragmented with the use of microTUBEs – 50 AFA Fiber Screw Cap (#520166, Covaris) and AFA-

BMJ Publishing Group Limited (BMJ) disclaims all liability and responsibility arising from any reliance placed on this supplemental material which has been supplied by the author(s)

Grade Water (#520101, Covaris). The following Covaris settings were used in order to perform the DNA fragmentation:

Holder	M220 Holder XTU (PN 500414)
Insert	M220 Holder XTU Insert microTUBE 50 µl (PN 500488)
Peak power	75 W
Duty factor	15 %
Cycles / burst	1000
Time of treatment	360 seconds
Temperature	20.0 Cº

To assure an optimal DNA fragmentation, sheared DNA fragments were evaluated with the High Sensitivity DNA Kit (#5067-4626, Agilent) in a Bioanalyzer equipment (Agilent), evaluating the peak size of the generated fragments. Next, an end repair and A-tailing to convert the 5' and 3' overhangs resulting from the DNA fragmentation into blunt ends was performed. The 3' to 5' exonuclease activity removed the 3' overhangs and the 5' to 3' polymerase activity filled in the 5' overhangs. The 3' ends became A-tailed during this reaction in order to prevent unwanted ligations. Following, it was carried out the adapter ligation, featuring the addition of Unique Molecular Indexes (UMIs) in order to identify unique molecules. After removing the excess of ligation reagents, each sample was indexed to identify each sequence during sequencing subsequent steps. Once DNA libraries were generated, the enrichment steps were performed. First, a hybridization-capture step (twice) was carried out, in which a pool of oligos specific to 523 genes hybridized to the prepared DNA libraries, which were later captured with the use of biotin probes that connected to streptavidin magnetic beads. Once the regions of interest were enriched, they were amplified. Following, libraries were quantified with the Qubit dsDNA HS Assay Kit (#Q32854, Invitrogen). A library concentration of at least 3

ng/µl was required to achieve an efficient bead-based library normalization. Quality control and library preparation details are shown in the **Supplemental Table S8**. Sequencing was carried out in a NextSeq 500 with the NextSeq 500/550 High Output Kit v2.5 reagents, using 300 cycles (High Output kit #20024908, Illumina). PhiX control V3 (#15017666, Illumina) was used as a sequencing control.

Oncomine Tumor Mutation Load Assay (TML) - 104 patients were sequenced with OTML. Four samples were excluded due to high deamination. Therefore, the total number of valid sequenced samples with OTML was 100. OTML targets 409 genes with a coverage of 1,65 Mb, again outperforming established requirements defined for TMB calculation. For library generation, 20 ng of DNA were used for the Oncomine™ Tumor Mutation Load Assay- Chef ready library preparation kit (Thermo Fisher Scientific A37910). Briefly, DNA was treated with 1 unit of Uracil-DNA Glycosylase (UDG) enzyme (Thermo Fisher Scientific 78310100UN) for 2 minutes at 37Cº followed by 10 minutes of heat inactivation at 50Cº. Treated DNA was diluted until 0.67 ng/ul and placed into an IonCode™ 96 Well PCR Plate (eight samples per plate). Library preparation was performed in the Ion Chef System with the following settings: 16 minutes of annealing/extension and 16 cycles.

Barcoded libraries were quantified with the Ion Library TaqMan® Quantitation Kit. According to kit instructions, each sample (including the standard and the negative control) was analysed in duplicate reactions and equal amounts of each library were combined before template preparation. As recommended for the Ion 540™ Chip, 8 libraries were combined at 33pM. Templating was done with the Ion Chef System and the chips were sequenced on the Ion S5 Sequencer. Quality control and library preparation details are shown in the **Supplemental Table S8**

Sample size of the study

Samples that surpassed quality requirements and were successfully analyzed with each of the assays are included in **Supplemental Table S1.** Due to several factors, explained on **Supplemental Figure S1** and **Supplemental Table S1**, the harmonization study was performed among the three panels with a final number of 96 samples.

Sequencing data analysis and TMB calculation

Illumina TruSight Oncology 500 (TSO500)- The bioinformatic analysis of the TSO500 sequencing data was performed with a DELL EMC Poweredge T640 server provided by Illumina. BCL files were transferred from the NextSeq 500 to the server. Then, an algorithm developed by Illumina (pipeline version 1.3.0.39) was utilized, receiving as a result an individual file per sample containing the TMB value (both Total and Non-Synonymous), the number of passing eligible variants, the number of passing eligible non synonymous variants and Microsatellite Instability data.

Oncomine Tumor Mutation Load Assay (OTML)- The bioinformatic analysis of the OTML sequencing data was performed with the Ion Reporter™ Software 5.10 analysis workflow. An average of 77 to 80 million reads were obtained for each chip, giving a mean coverage depth of 550X. With Ion Reporter™ Software 5.10 the automatic variant calling was carried out, applying a filter of a minimum of 5% allelic frequency and 60x coverage depth. For samples with a high estimation of Single Nucleotide Polymorphism (SNP) proportion (>20) consistent with deamination (C to T) the filter was increased to 10% allelic frequency. With these parameters adjusted, TMB was calculated as the total number of somatic mutations, synonymous and non-synonymous, divided by the number

of exonic bases with sufficient coverage and multiplied by 106 to obtain it per Megabase. If TMB was higher than 25 then it was necessary to introduce a correction element multiplying by a calibration factor (calibration slope + 25).

PD-L1 Immunohistochemistry (IHC) and scoring

FFPE sections were stained with anti-PD-L1 22C3 mouse monoclonal primary antibody by utilizing the EnVision FLEX visualization system on a Dako Autostainer Link 48 system with negative control reagents and cell line run controls as described in the PD-L1 IHC 22C3 pharmDx package insert[4]. Two comparison groups were selected based on PD-L1 expression: PD-L1<1% (N=55) versus PDL1 ≥1% (N=41).Results of the TMB values obtained for each group and demographic comparisons among the groups are shown in **Supplemental Table S2**.

Inter-laboratory comparison

A total of three institutions participated in the study to perform the comprehensive genomic profiling with TSO500 and OTML and to carry out inter-center reproducibility assays of the panels used. TruSight Oncology 500 panel (TSO500 Illumina) was performed at the i+12 Research Institute / 12 de Octubre University Hospital (Institution 1), under an Illumina Early Access Agreement. Oncomine Tumor Mutational Load (OTML, Thermo Fisher) panel was performed in HM Sanchinarro University Hospital (Institution 2). For the inter-institutional cross-validations, 25% of samples were cross-evaluated by a different institution. Cross-validation of the Oncomine TML panel was performed at the imas12 Research Institute / University Hospital 12 de Octubre. Cross-validation of TSO500 was carried out at Ramón y Cajal University Hospital (Institution 3). Details are shown in **Supplemental Table S3**.

Statistical Analyses

All graph and results were generated using Stata 16 software[5]. The concord[6]kappa, cutpt[7]and rocregplot packages were used. Measurements of agreement among the three methods were evaluated using the concordance correlation coefficient[8, 9]. The concordance correlation coefficient combines measures of both precision and accuracy to determine how far the observed data deviate from the line of perfect concordance (that is, the line at 45 degrees on a square scatterplot). The bias between two methods was estimated with the mean of the difference between the two measurements per subject. The 95% limits of agreement[10]were constructed with the size of difference, bias, and the standard deviation of the differences. The relationship between difference and mean was plotted in terms absolute (difference between measures for each subject) and relative (difference between measures divide by mean of measures for each subject) with the limits of agreement. Using the cutpoint values of 10, 13, 16 and 20 for the three methods, the agreement between dichotomy measures was estimated using the kappa statistic with 95% confidence intervals. The ability discriminatory of each method (TSO500 and OTML), with respect to FO method, reference assay, was studied using the area under the receiver operating characteristic curve (ROC). The decision thresholds were displayed on the ROC plot and the adjusted cutpoint[11] was estimated for each method. The sensitivity, specificity, positive and negative predictive values, positive and negative likelihood ratios, and accuracy were estimated to characterize the different cut-off points. Details are shown in **Supplemental Table S4.**

Immune markers immunohistochemistry (IHC), scoring and statistical analysis

Formalin-fixed paraffin-embedded (FFPE) tumor samples were sectioned at 3,5 µm and mounted in BOND Plus Slides (#S21.2112.A, Leica). The protocol consisted on epitope retrieval and staining (antibody incubation, peroxide block, post primary, mixed Diaminobenzidine and hematoxilin) and was carried out in a BOND-III IHQ-ISH Automated Station (Leica). Primary antibodies used were: CD20 (DAKO M0755 1/50), CD4 (Leica NCL-L-CD4-368 1/50), CD68 (DAKO M0814 1/1000) and CD8 (DAKO M7103 1/19). Primary antibodies were diluted in Primary Antibody Diluent, (#AR9352, Leica). For antigen retrieval, Epitope Retrieval Solution 1 (ER1, #AR9961, Leica) and Epitope Retrieval Solution 2 (ER2, #AR9640, Leica) were used. Specific retrieval conditions for each marker were ER1 buffer (pH6) fof CD20, CD68 and CD8 and ER2 buffer (pH9 for CD4. Retrieval times were 20 min for CD20 and CD4, 10 min for CD68 and 30 min for CD8. Incubation time was 20 min for all.Visualization was performed with the BOND Polymer Refine Detection (#DS9800-CN, Leica), which uses Diaminobenzidine (DAB) as the chromogen to detect the marker and haematoxylin as a counterstain. Positive staining for each maker was confirmed in parallel using human tonsil tissue.

Three comparison groups of samples were selected based on the expression of these markers with a score: Score 0 (<1% of expression), Score 1 (1-10% of expression) and Score 2 (>10% of expression). Results of the TMB values obtained for each group with the three assays and information regarding the level of expression of the markers can be found in **Supplemental Table S5**.

A Kruskal-Wallis test (non-parametric) test was carried out to evaluate differences in the TMB values among the groups with different levels of expression of the markers CD20, CD4, CD8 and CD68. The multiple comparison analysis was performed with a Dunn's test.

Patient and public involvement statement

Not required

REFERENCES

1. Frampton GM, Fichtenholtz A, Otto GA, et al. Development and validation of a clinical cancer genomic profiling test based on massively parallel DNA sequencing. *Nat Biotechnol*. 2013;31:1023-31. doi:10.1038/nbt.2696

2. FoundationOne CDx FDA Approval, 2017. Available at: https://www.accessdata.fda.gov/cdrh_docs/pdf17/P170019a.pdf.

3. Chalmers ZR, Connelly CF, Fabrizio D, et al. Analysis of 100,000 human cancer genomes reveals the landscape of tumor mutational burden. *Genome Med*. 2017;9:34. doi: 10.1186/s13073-017-0424-2.

4. Roach C, Zhang N, Corigliano E, et al. Development of a Companion Diagnostic PD-L1 Immunohistochemistry Assay for Pembrolizumab Therapy in Non-Small-cell Lung Cancer. *Appl Immunohistochem Mol Morphol*. 2016;24:392-7. doi: 10.1097/PAI.0000000000000408.

5. StataCorp. 2019. Stata Statistical Software: Release 16. College Station, TX: StataCorp LLC.

BMJ Publishing Group Limited (BMJ) disclaims all liability and responsibility arising from any reliance placed on this supplemental material which has been supplied by the author(s)

6. Steichen TJ, N.J. Cox. A note on the concordance correlation coefficient. *Stata Journal*; 2002. https://doi.org/10.1177/1536867X0200200206

7. Clayton P. CUTPT: Stata module for empirical estimation of cutpoint for a diagnostic test. Statistical Software Components S457719, Boston College Department of Economics; 2013.

8. Lin LI. A concordance correlation coefficient to evaluate reproducibility. *Biometrics*. 1989;45:255-68. doi: 10.2307/2532051

9. Lin. A note on the concordance correlation coefficient. *Biometrics*; 2000.

10. Bland JM, Altman DG. Statistical methods for assessing agreement between two methods of clinical measurement. *Lancet*. 1986;1:307-10.

11. Liu X. Classification accuracy and cut point selection. *Stat Med.* 2012;31:2676-86. doi: 10.1002/sim.4509.

SUPPLEMENTAL FIGURES AND TABLES

Supplemental Figure S1. Flowchart of the project

Supplemental Figure S2. Side-by-side comparisons of the part of the cohort whose correlations were made against Foundation One panel (FO; *N*=52) and the part of the cohort whose correlations were made against Foundation One CDx (F1CDx;

***N*=44). A, B.** Linear regression of TMB results obtained with TSO500 versus FO/F1CDx. **C, D.** Linear regression of TMB results obtained with OTML compared to those obtained with FO/F1CDx.

Supplemental Figure S3. Quantification of the degree of agreement among TMB determination methods in the group of tumors expressing levels of PD-L1<1%. A-C. Comparison between TSO500 and OTML. **D-F.** Comparison between Foundation One (FO) and TSO500; **G-I.** Comparison between FO and OTML; **A,B,D,E,G,H**. Bland Altman plots. **C,F,I.** Degree of agreement shown by the average difference, standard deviation, 95% limits of agreement and Concordance Correlation Coefficient. TMB was calculated as total (synonym plus non-synonym mutations per Megabase of DNA) in a cohort of N=55 early stage NSCLC tumors with PD-L1<1%.

Supplemental Figure S4. Quantification of the degree of agreement among TMB determination methods in the group of tumors expressing levels of PD-L1≥1%. A-C. Comparison between TSO500 and OTML. **D-F.** Comparison between Foundation One (FO) and TSO500; **G-I.** Comparison between FO and OTML; **A,B,D,E,G,H**. Bland Altman plots. **C,F,I.** Degree of agreement shown by the average difference, standard deviation, 95% limits of agreement and Concordance Correlation Coefficient. TMB was calculated as total (synonym plus non-synonym mutations per Megabase of DNA) in a cohort of N=41 early stage NSCLC tumors with PD-L1≥1%.

Supplemental Figure S5. Visual distribution of the TMB values obtained with TSO500 or OTML for samples categorized as TMB$^{med\text{-}lo}$ with Foundation One, using

different cut-off values. A, B. Samples are selected in the $TMB^{med\text{-}lo}$ category based on their total TMB data obtained by Foundation One and selected with four different cut-offs: TMB^{total} < 10 (**A**, *N*=47), < 13 (**B**, *N*=62), < 16 (**C**, *N*=69) or < 20 (**D**, *N*=79) muts/Mb, respectively. Only samples below the selected threshold with Foundation One (samples in pink) are additionally plotted in the same graph for TSO500 and OTML, in order to visualize the distribution of TMBs with the assays under study. TMB values obtained with TSO500 (green) and OTML (blue) are shown. Several patients that are categorized as $TMB^{med\text{-}lo}$ using the standard method would have been labelled as TMB^{high} with TSO500 and/or OTML, if keeping the same cutoff value.

Supplemental Figure S6. Tumor Mutation Burden determined by three NGS panels versus immune infiltration of $CD4^+$ T cells, $CD8^+$ T cells, $CD20^+$ B cells and $CD68^+$ macrophages in NSCLC patient samples.
A-D. Immunohistochemical analyses of $CD8^+$ (**A**), $CD4^+$ (**B**), $CD20^+$ (**C**) and $CD68^+$ (**D**) immune infiltrates (% of cell infiltrate in the tumor) versus the number of mutations per megabase evaluated by Foundation One test in N=96 FFPE NSCLC samples. **E-H.** Immunohistochemical analyses of $CD8^+$ (**E**), $CD4^+$ (**F**), $CD20^+$ (**G**) and $CD68^+$ (**H**) immune infiltrates (% of cell infiltrate in the tumor) versus the number of mutations per megabase evaluated by TSO500 test in N=96 FFPE NSCLC samples. **I-L.** Immunohistochemical analyses of $CD8^+$ (**I**), $CD4^+$ (**J**), $CD20^+$ (**K**) and $CD68^+$ (**L**) immune infiltrates (% of cell infiltrate in the tumor) versus the number of mutations per megabase evaluated by OTML test in N=96 FFPE NSCLC samples

Supplemental Table S1. Sequencing results and TMB determined with the two NGS panels under study and the reference standard method Foundation One. Comparison of the obtained results among the three methods (*N*=96).

Supplemental Table S1-A: TMB determined with TSO500 (*N*=104)

Supplemental Table S1-B: TMB determined with OTML (*N*=104)

Supplemental Table S1-C: TMB determined with FO/F1CDx (*N*=110)

Supplemental Table S1-D: Comparison of the determined TMB among the three methods with common samples (*N*=96)

Supplemental Table S1-E: Samples evaluated per assay

Supplemental Table S1-F: Descriptive analysis of the data obtained with the three methods.

Supplemental Table S1-G: Evaluation of the concordance correlation coefficient (Bland Altman)

Supplemental Table S2. TMB determination with the three NGS panels used in different PD-L1 expressing samples.

Supplemental Table S2-A: Tumor expression of PD-L1 measured by IHC and values of total TMB calculated with the three panels for each sample.

Supplemental Table S2-B: Comparison of the demographic characteristics among the groups of patients with different expression of PD-L1 (*N*=96).

Supplemental Table S3. Results from reproducibility assays: TMB^{total} results obtained in the inter-institution reproducibility assays.

Supplemental Table S3-A: Reproducibility assay TMB results for TSO500 (*N*=21) between Institution 1 and Institution 3.

BMJ Publishing Group Limited (BMJ) disclaims all liability and responsibility arising from any reliance placed on this supplemental material which has been supplied by the author(s)

Supplemental Table S3-B: Reproducibility assay TMB results for OTML (*N*=23) between Institution 1 and Institution 2.

Supplemental Table S4. ROC (Receiver Operating Characteristic) curves for the determination of the adjusted cutoff values with TSO500 (P2) and OTML (P3), compared to fixed values selected for Foundation One (P1) as reference method.

Supplemental Table S4-A: P1(10)P2 – ROC curve of TSO500 for cutoff 10 muts/Mb by FO

Supplemental Table S4-B: P1(10)P3 – ROC curve of OTML for cutoff 10 muts/Mb by FO

Supplemental Table S4-C: P1(13)P2 – ROC curve of TSO500 for cutoff 13 muts/Mb by FO

Supplemental Table S4-D: P1(13)P3 – ROC curve of OTML for cutoff 13 muts/Mb by FO

Supplemental Table S4-E: P1(16)P2 – ROC curve of TSO500 for cutoff 16 muts/Mb by FO

Supplemental Table S4-F: P1(16)P3 – ROC curve of OTML for cutoff 16 muts/Mb by FO

Supplemental Table S4-G: P1(20)P2 – ROC curve of TSO500 for cutoff 20 muts/Mb by FO

Supplemental Table S4-H: P1(20)P3 – ROC curve of OTML for cutoff 20 muts/Mb by FO

Supplemental Table S4-I: Differences among ROC curves

Supplemental Table S4-J: Empiric optimal cut-offs for TSO500 and OTML equivalent to cut-off values of 10, 13, 16 and 20 muts/Mb by FO

Supplemental Table S4-K: Adjusted cut-offs for TSO500 and OTML equivalent to cut-off values of 10, 13, 16 and 20 muts/Mb by FO with a sensitivity >90%

Supplemental Table S5. TMB value distribution according to the level of infiltration of several immune cell subpopulations evaluated by immunohistochemistry.

Supplemental Table S6. Demographic information of the cohort and its comparative statistical analysis according to TMB value.

Supplemental Table S6-A: Demographic information of the cohort (N=110 and N=96)

Supplemental Table S6-B: Comparison of the demographic characteristics between the TMB^{high} and $TMB^{med/low}$ groups of patients, as identified by Foundation One using a cutoff of 10 muts/Mb (N=96)

Supplemental Table S7. Characteristics of the NGS panels used in the TMB^2 Harmonization study. Technological comparison among TruSight Oncology 500 (TSO500), Oncomine Tumor Mutation Load (OTML) and Foundation One (FO)

Supplemental Table S8. Quality control and input parameters of library generation with each of the three NGS methods used for the TMB^2 Harmonization study and inter-institution reproducibility assays.

Supplemental Table S8-A: Quality Control parameters and library generation data of TSO500 (*N*=104)

Supplemental Table S8-B: Quality Control parameters and library generation data of OTML (*N*=104)

Supplemental Table S8-C: Quality Control parameters and library generation data of FO/F1CDx (*N*=110)

Supplemental Table S8-D: Quality control parameters and library generation data of inter-institutions reproducibility assay for TSO500 (*N*=21)

Supplemental Table S8-E: Quality control parameters and library generation data of inter-institutions reproducibility assay for OTML (*N*=23)

Ramos-Paradas J, *et al. J Immunother Cancer* 2021; 9:e001904. doi: 10.1136/jitc-2020-001904

Supplementary Figure S1

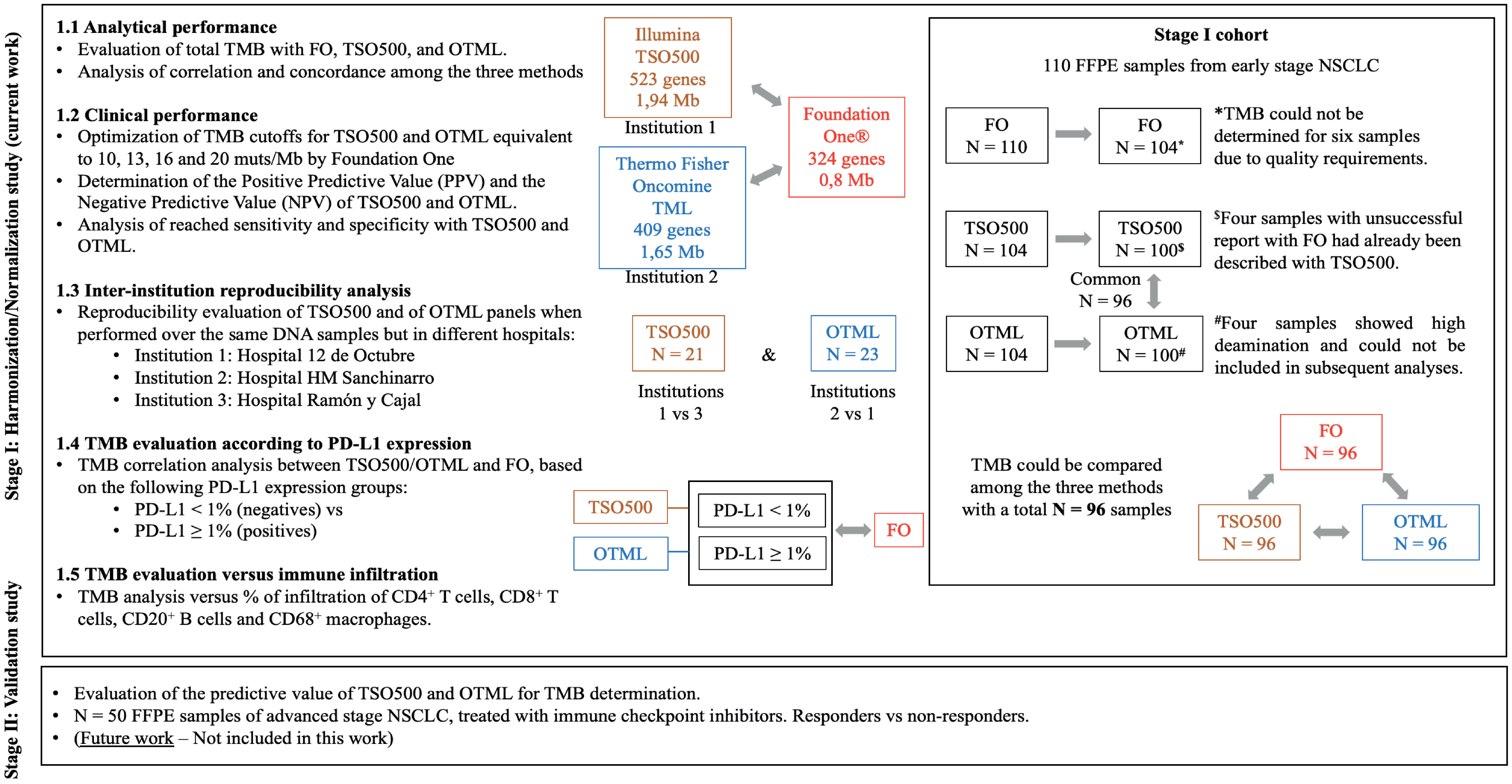

Supplementary Figure S2

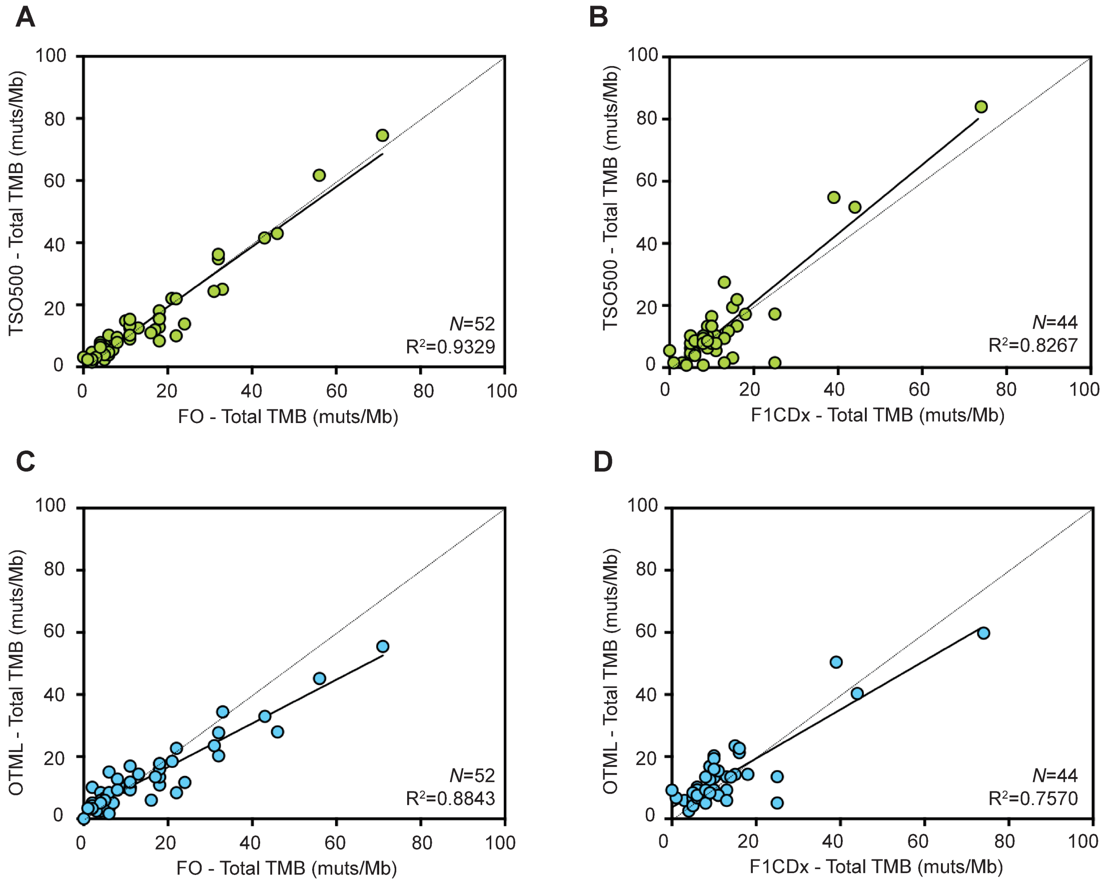

Supplementary Figure S3

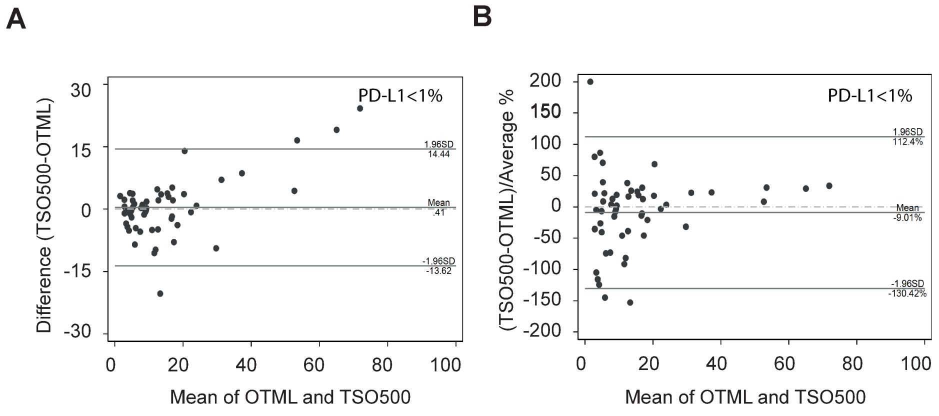

C

Difference average	0.414
Std Dev	7.159
95% Limits of agreement	-13.617<br>14.445
Concordance Correlation Coefficient	0.896
CI (95%)	[0.854; 0.937]

TSO500 vs OTML

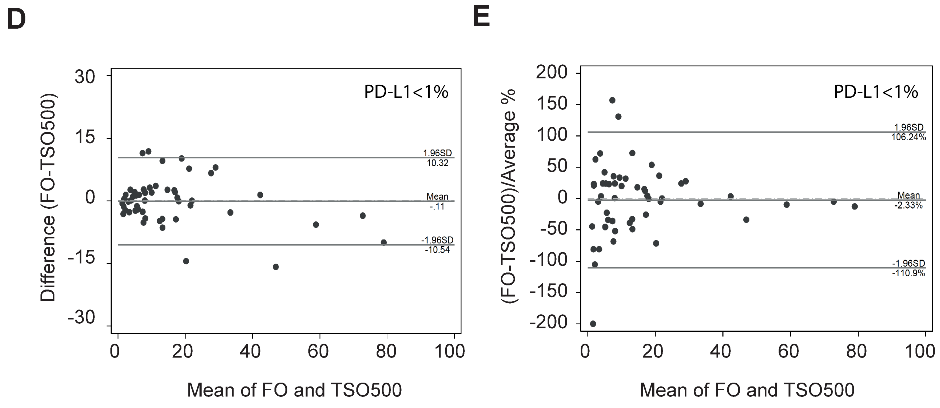

F

Difference average	-0.111
Std Dev	5.322
95% Limits of agreement	-10.542<br>10.319
Concordance Correlation Coefficient	0.951
CI (95%)	[0.926; 0.975]

FO vs TSO500

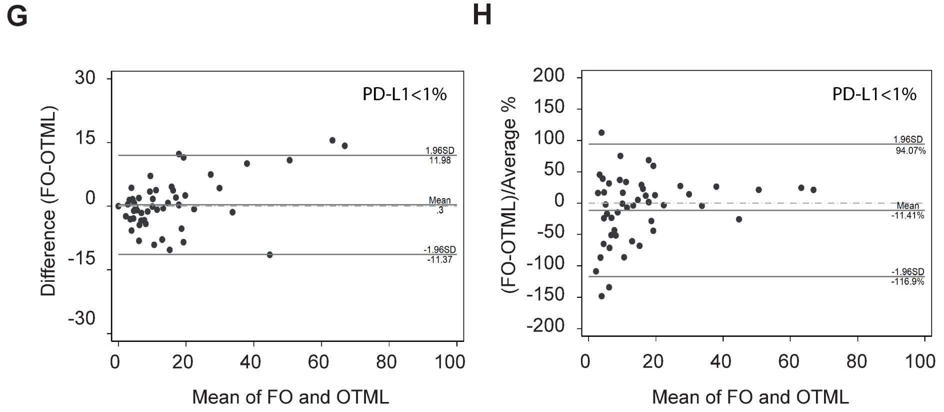

I

Difference average	0.302
Std Dev	5.958
95% Limits of agreement	-11.375<br>11.979
Concordance Correlation Coefficient	0.919
CI (95%)	[0.882; 0.956]

FO vs OTML

Supplementary Figure S4

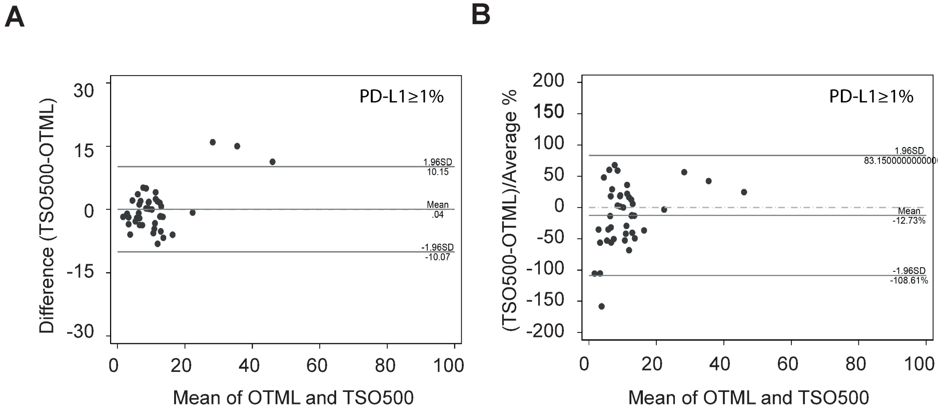

C

Difference average	0.038
Std Dev	5.159
95% Limits of agreement	-10.074<br>10.149
Concordance Correlation Coefficient	0.832
CI (95%)	[0.755; 0.909]

TSO500
vs OTML

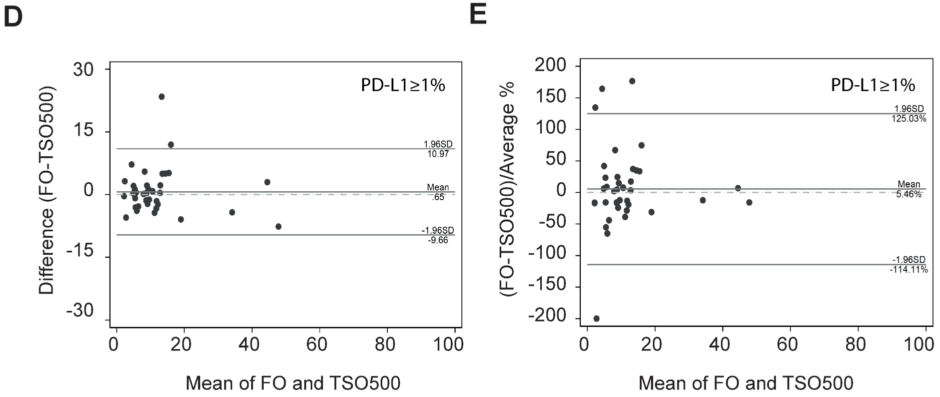

F

Difference average	-0.653
Std Dev	5.263
95% Limits of agreement	-9.662<br>10.969
Concordance Correlation Coefficient	0.861
CI (95%)	[0.781; 0.942]

FO
vs TSO500

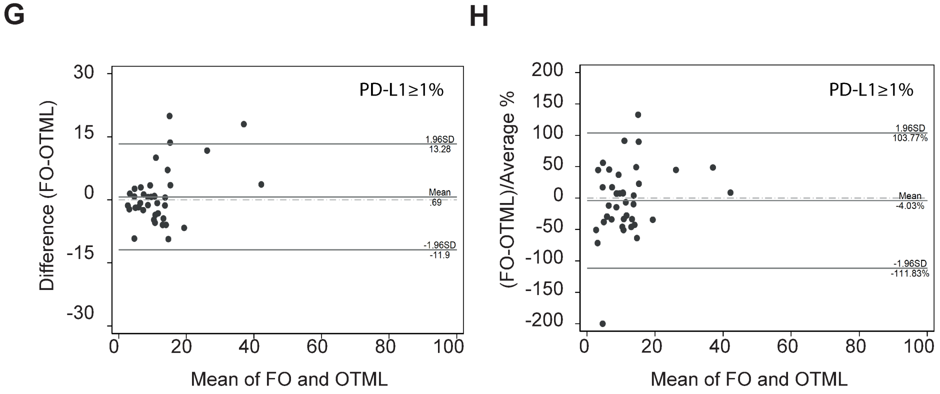

I

Difference average	0.691
Std Dev	6.423
95% Limits of agreement	-11.897<br>13.279
Concordance Correlation Coefficient	0.722
CI (95%)	[0.586; 0.858]

FO
vs OTML

Supplementary Figure S5

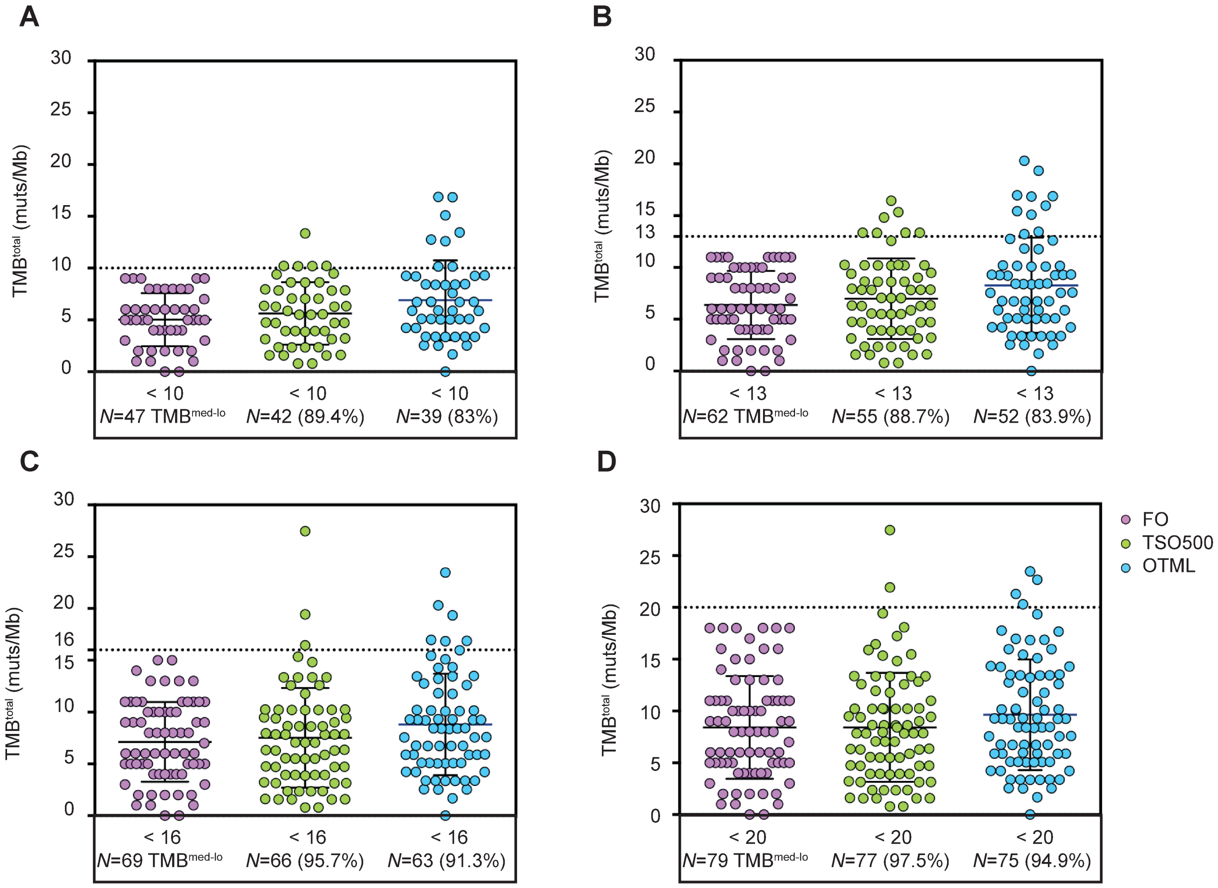

Supplementary Figure S6

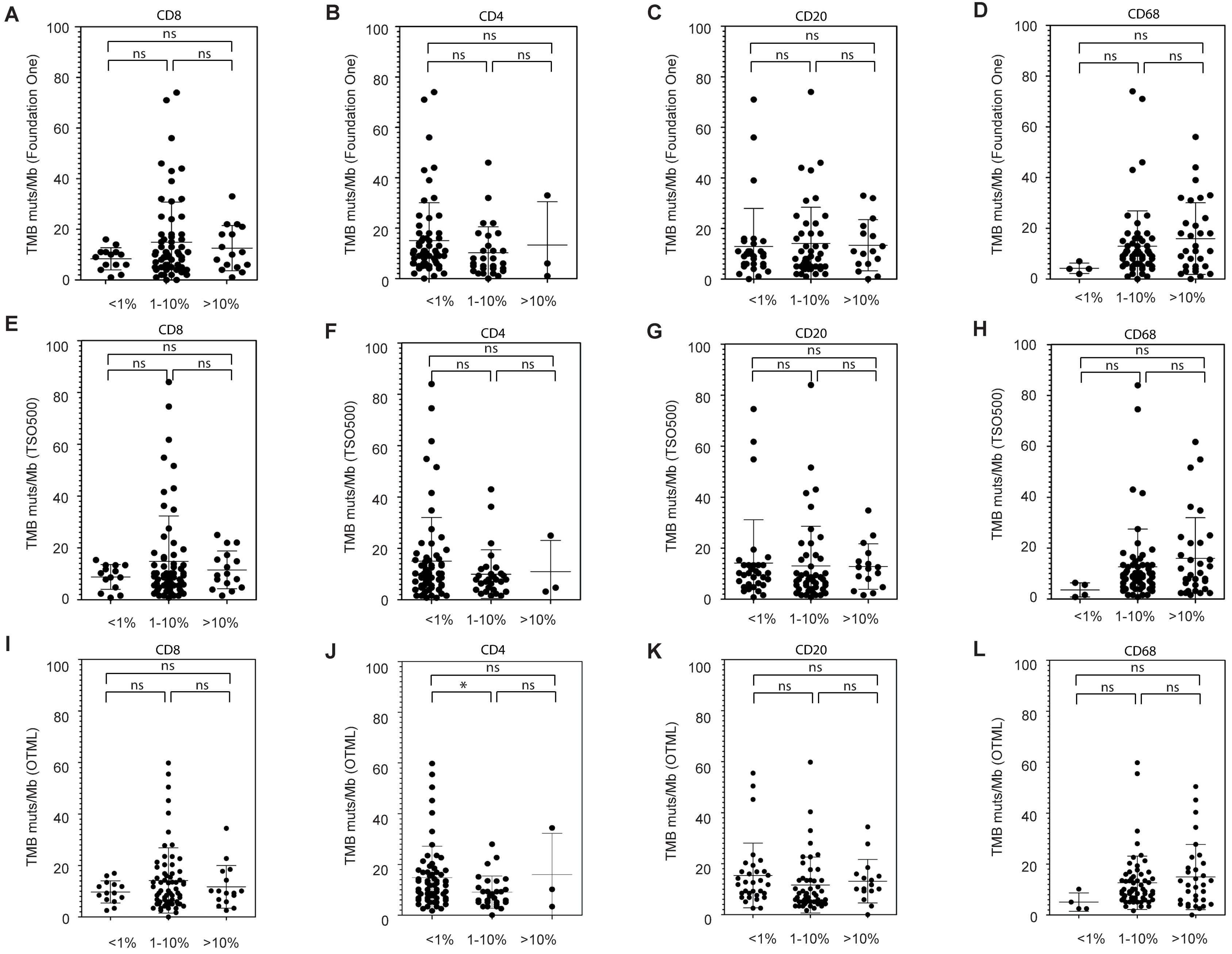

Supplement: Supplementary data [file jitc-2020-001904supp001.pdf]
